# Seeing the Unseen: A Rare Ocular Complication of Tuberculous Meningoencephalitis

**DOI:** 10.7759/cureus.108975

**Published:** 2026-05-16

**Authors:** Rathod Saiharshini, Yatri Patel, Amit Pal Singh, Minakshi Dhar, Monika Pathania

**Affiliations:** 1 Geriatric Medicine, All India Institute of Medical Sciences, Rishikesh, IND; 2 Internal Medicine, All India Institute of Medical Sciences, Rishikesh, IND

**Keywords:** bilateral oculomotor nerve palsy, bilateral ptosis, hydrocephalus, ophthalmoplegia, tuberculous meningoencephalitis

## Abstract

Tuberculous meningitis (TBM) is the most severe form of central nervous system (CNS) tuberculosis and carries significant morbidity, particularly when diagnosis is delayed. Cranial nerve involvement is a recognized complication, most frequently affecting the abducens nerve (CN VI); bilateral oculomotor nerve (CN III) palsy, however, is exceedingly rare and typically signifies pathology at the level of the midbrain. The oculomotor nerve originates from paired nuclei in the midbrain tegmentum at the level of the superior colliculus; any compressive or inflammatory lesion at this site can produce bilateral CN III deficits. We report a 70-year-old man with type 2 diabetes mellitus presenting with subacute fever, headache, and altered sensorium, who subsequently developed bilateral ptosis with ophthalmoplegia. The brain MRI demonstrated multiple disseminated tuberculomas with a focal midbrain lesion at the oculomotor nuclear-fascicular complex and communicating hydrocephalus. This case highlights an uncommon neuro-ophthalmological manifestation of TBM and emphasizes the critical role of early clinico-radiological correlation in diagnosis and management.

## Introduction

Tuberculosis continues to pose a significant health burden in endemic regions, with central nervous system (CNS) involvement representing one of its most devastating manifestations. Tuberculous meningitis (TBM) is associated with substantial neurological morbidity, particularly when diagnosis is delayed [1]. Cranial neuropathies are well-recognized complications, resulting from inflammatory exudates at the base of the brain, most frequently involving the abducens nerve (CN VI), followed by the facial and oculomotor nerves [1].

The neuroanatomical basis of third nerve involvement in TBM warrants brief consideration. The oculomotor nerve nuclei reside in the midbrain tegmentum at the level of the superior colliculus, adjacent to the cerebral aqueduct. The nerve's fascicles traverse the red nucleus and cerebral peduncle before exiting the interpeduncular fossa. Bilateral CN III dysfunction, therefore, implies either simultaneous injury to both nuclei or fascicles within the midbrain or, less commonly, involvement of both nerves along their cisternal course within the basal exudates. The diagnostic significance of bilateral third nerve palsy in TBM is considerable: it is a rare finding that strongly suggests parenchymal or brainstem pathology beyond simple meningeal irritation and demands urgent structural neuroimaging [2,3].

Although oculomotor nerve involvement has been described in TBM, bilateral third nerve palsy remains exceedingly uncommon. Previous studies have reported atypical cranial nerve involvement in tuberculosis [2,3]. The present case is distinctive in demonstrating a clear radiological correlation between a focal midbrain tuberculoma and bilateral ophthalmoplegia, providing a direct structural explanation for this rare neuro-ophthalmological manifestation.

## Case presentation

Clinical timeline and presentation

A 70-year-old man with known type 2 diabetes mellitus presented to the emergency department following a three-week history of low-grade fever, persistent bifrontal headache, and progressive alteration in sensorium. The illness evolved subacutely: headache onset preceded hospitalization by approximately three weeks, with progressive worsening in intensity over the subsequent two weeks, associated with nausea and non-projectile vomiting. Bilateral ptosis was first noted approximately one week prior to admission, with progressive restriction of eye movements becoming apparent in the days immediately before presentation. There was no history of prior tuberculous disease, known contact with tuberculosis, or recent immunosuppressive therapies. He was not on anti-tubercular treatment and had not received a Bacille Calmette-Guérin (BCG) vaccination in adulthood.

Examination findings

**Figure 1:**
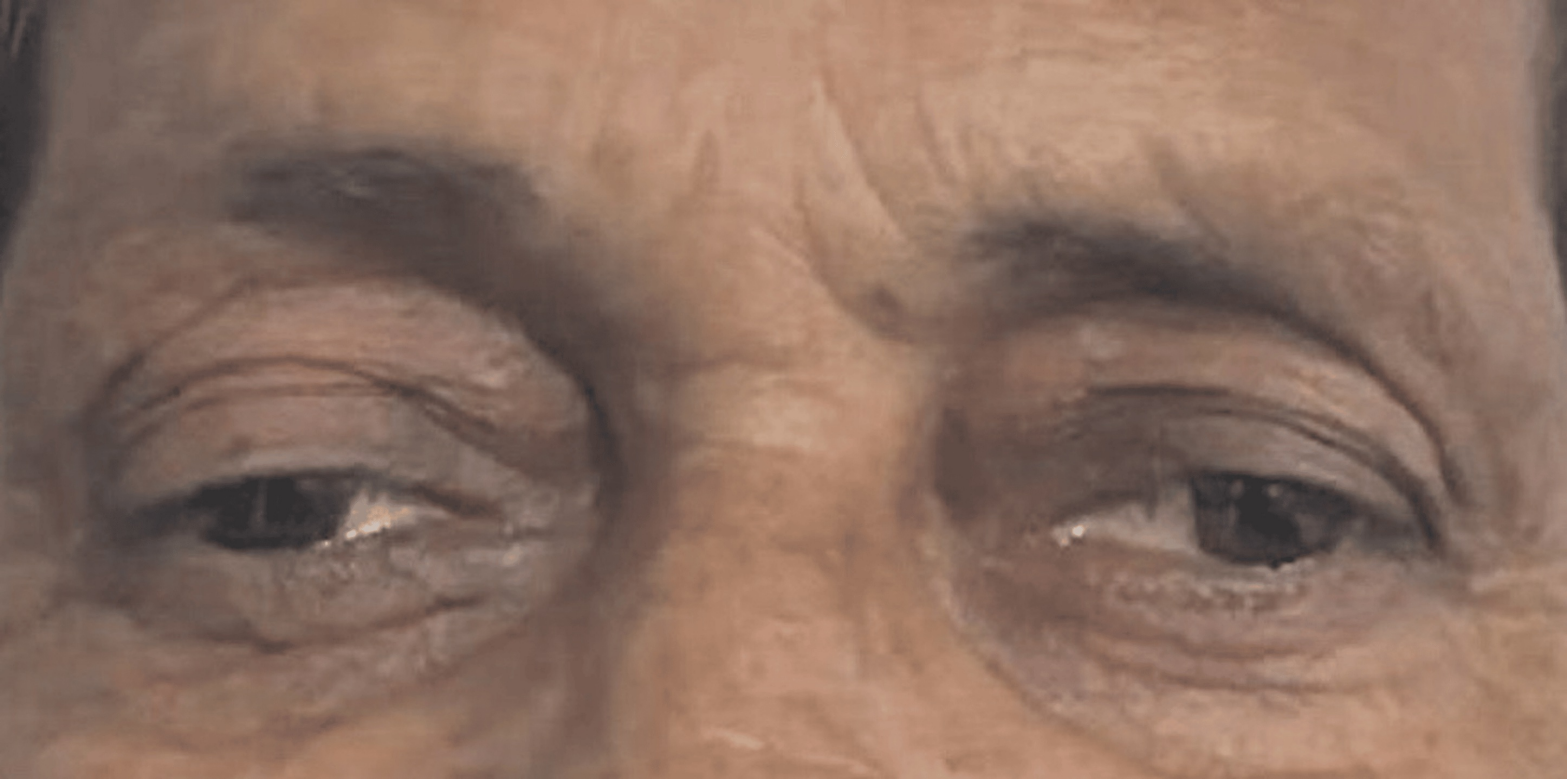
Bilateral ptosis, more pronounced on the left side

**Figure 2:**
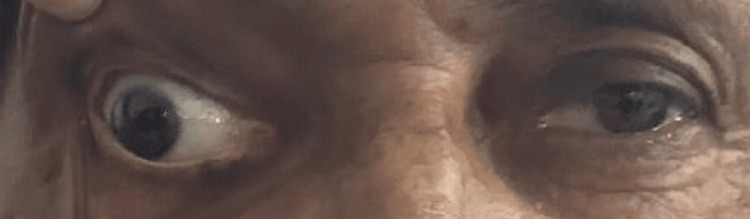
Left eye positioned "down and out" (exotropia with slight depression), consistent with complete left third nerve palsy

On general examination, the patient was febrile (38.4°C), tachycardic (102/minute), and normotensive. Neurological assessment revealed a Glasgow Coma Scale (GCS) score of E4V4M5 (13/15) [4], with disorientation to time, place, and person. Meningeal signs were present, with neck stiffness and a positive Kernig's sign. Cranial nerve examination demonstrated bilateral ptosis, more pronounced on the left (Figure 1). The left eye was positioned down and out with restricted adduction, elevation, and depression, consistent with a complete left third cranial nerve palsy (Figure 2). The right eye showed mild ptosis with limitation of adduction and upward gaze. Notably, both pupils were equal and reactive to light (4 mm bilaterally), indicating pupil-sparing bilateral oculomotor involvement, a finding discussed further below. The remaining cranial nerve examination was unremarkable. Motor, sensory, cerebellar, and long tract examinations were intact.

Laboratory investigations are summarized in Table 1.

**Table 1:** Laboratory and CSF investigations CSF: cerebrospinal fluid, ADA: adenosine deaminase, CBNAAT: cartridge-based nucleic acid amplification test, ESR: erythrocyte sedimentation rate, HIV: human immunodeficiency virus, HbA1c: hemoglobin A1c

Investigation (units)	Observed value	Reference range
Hemoglobin (g/dL)	10.2	13.0-17.0
Total leukocyte count (/µL)	11,400	4,000-11,000
Platelet Count (×10³/µL)	210	150-400
ESR (mm/hour)	78	0-20
Random blood sugar (mg/dL)	186	70-140
HbA1c (%)	8.2	<5.7
Serum sodium (mEq/L)	132	136-145
Serum potassium (mEq/L)	3.9	3.5-5.1
Serum creatinine (mg/dL)	1.1	0.7-1.2
Serum albumin (g/dL)	2.8	3.5-5.0
C-reactive protein (mg/L)	64	<10
HIV serology	Non-reactive	Non-reactive
CSF appearance	Turbid, yellowish	Clear, colorless
CSF opening pressure (mmHg)	260	70-180
CSF protein (mg/dL)	148	15-45
CSF glucose (mg/dL)	32	50-80
CSF:serum glucose ratio	0.17	>0.5
CSF cell count (/µL)	120 (lymphocytic predominance)	-
CSF ADA (U/L)	22	<10
CSF CBNAAT (Xpert MTB/RIF)	Negative	Negative

Neuroimaging

**Figure 3:**
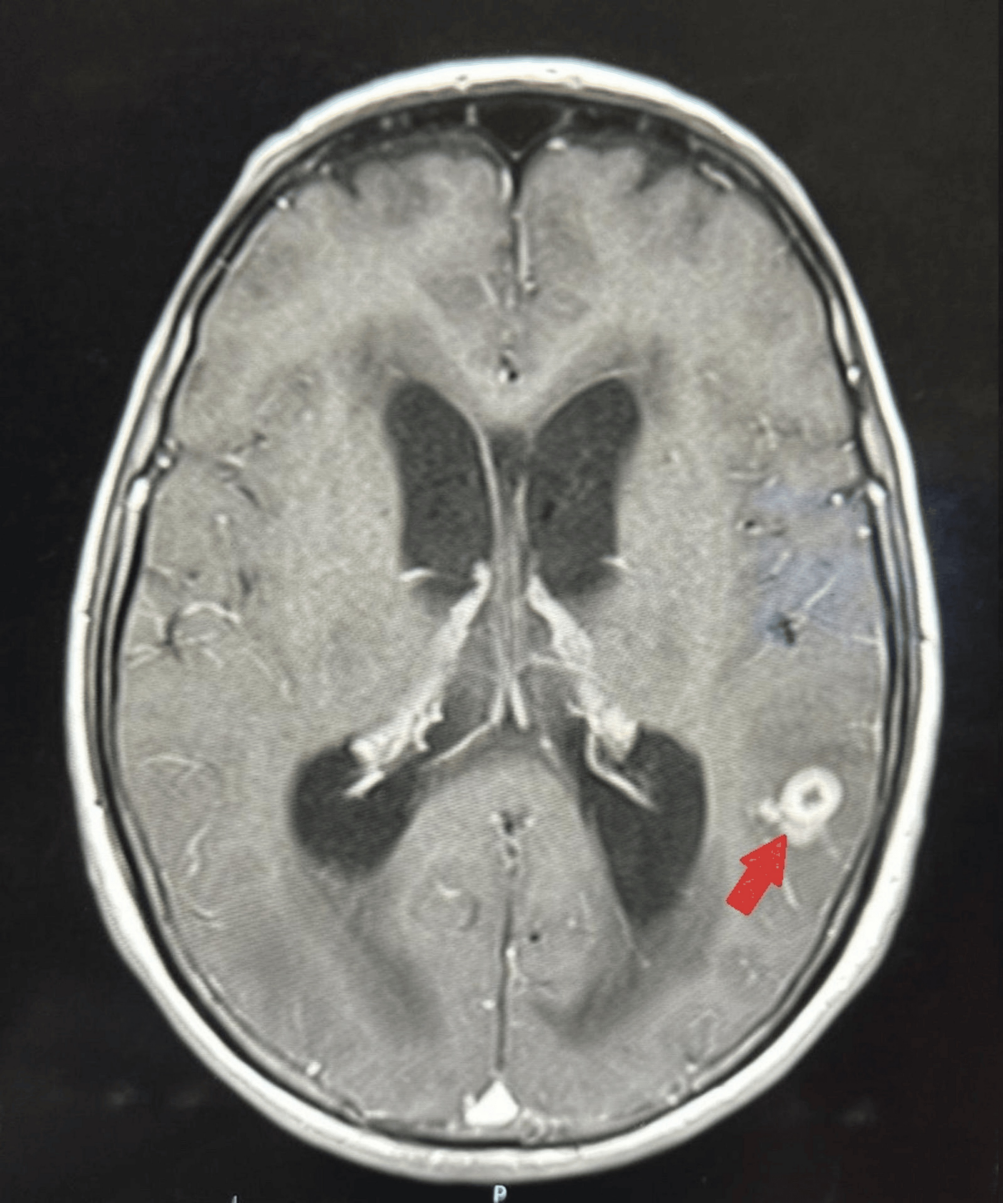
T1-weighted image: communicating hydrocephalus with ventricular dilatation (red arrow)

**Figure 4:**
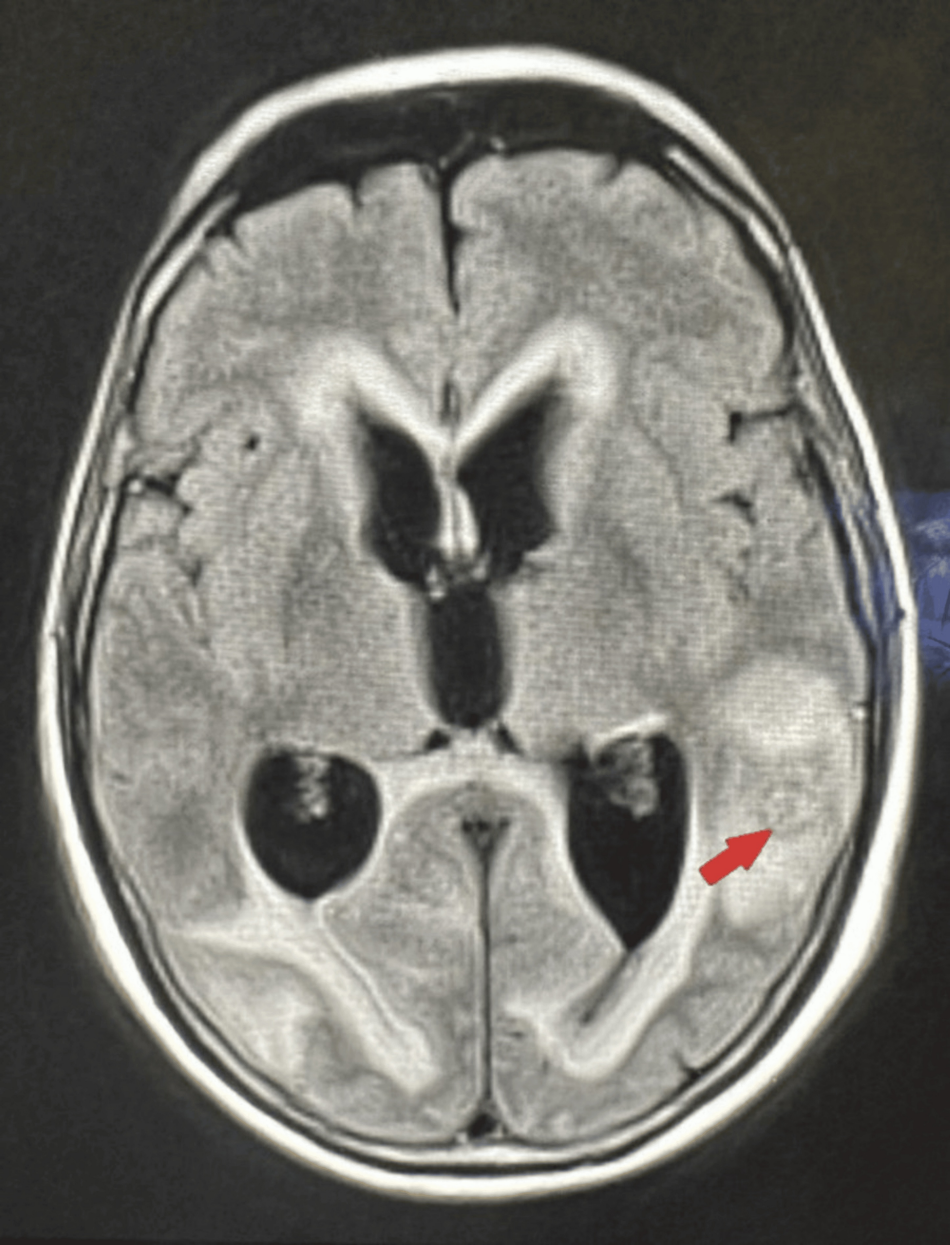
T2/FLAIR: periventricular hyperintensities, suggestive of trans-ependymal CSF seepage (red arrow) FLAIR: fluid-attenuated inversion recovery, CSF: cerebrospinal fluid

**Figure 5:**
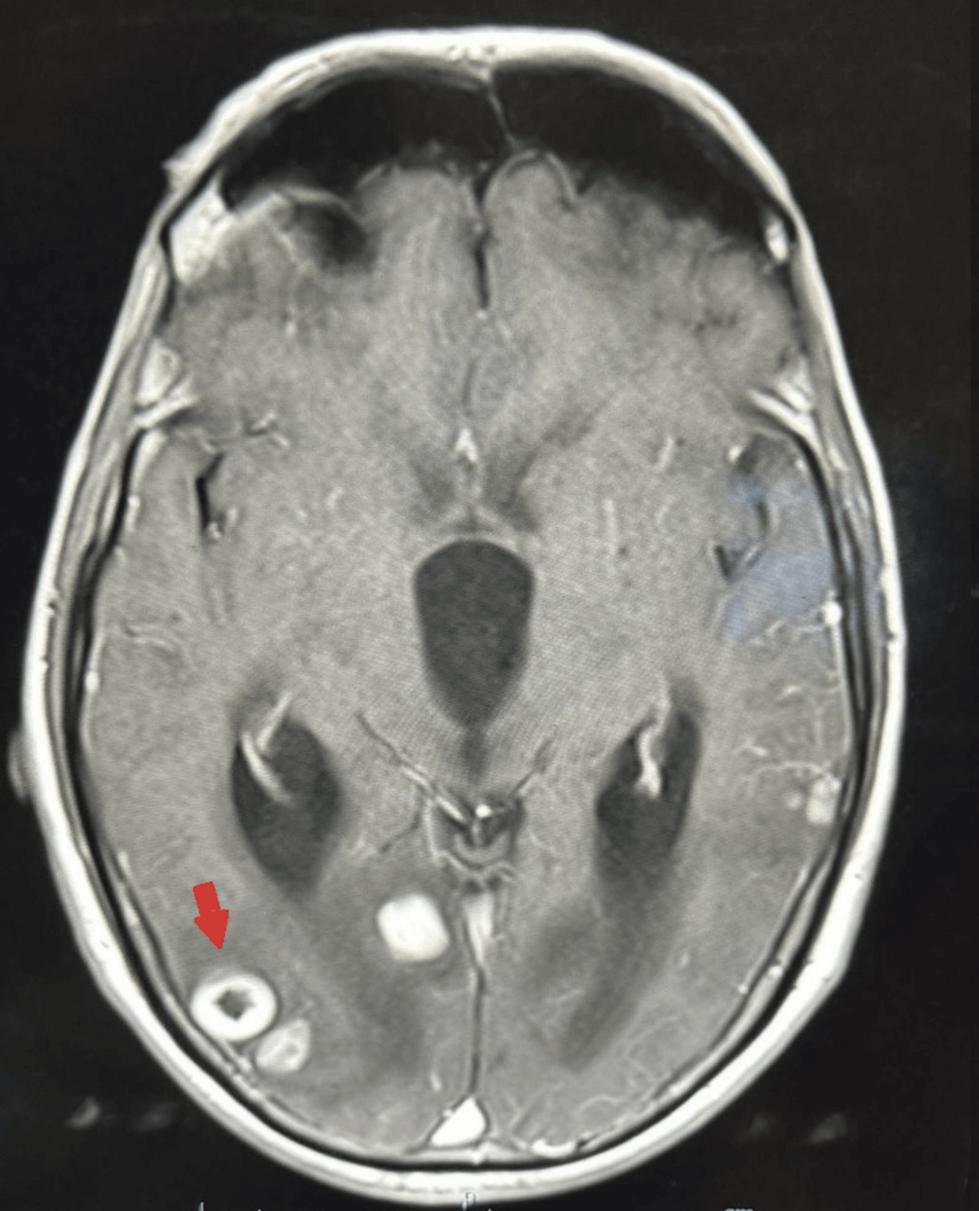
FLAIR: ring lesion with surrounding edema, consistent with tuberculoma (red arrow) FLAIR: fluid-attenuated inversion recovery

**Figure 6:**
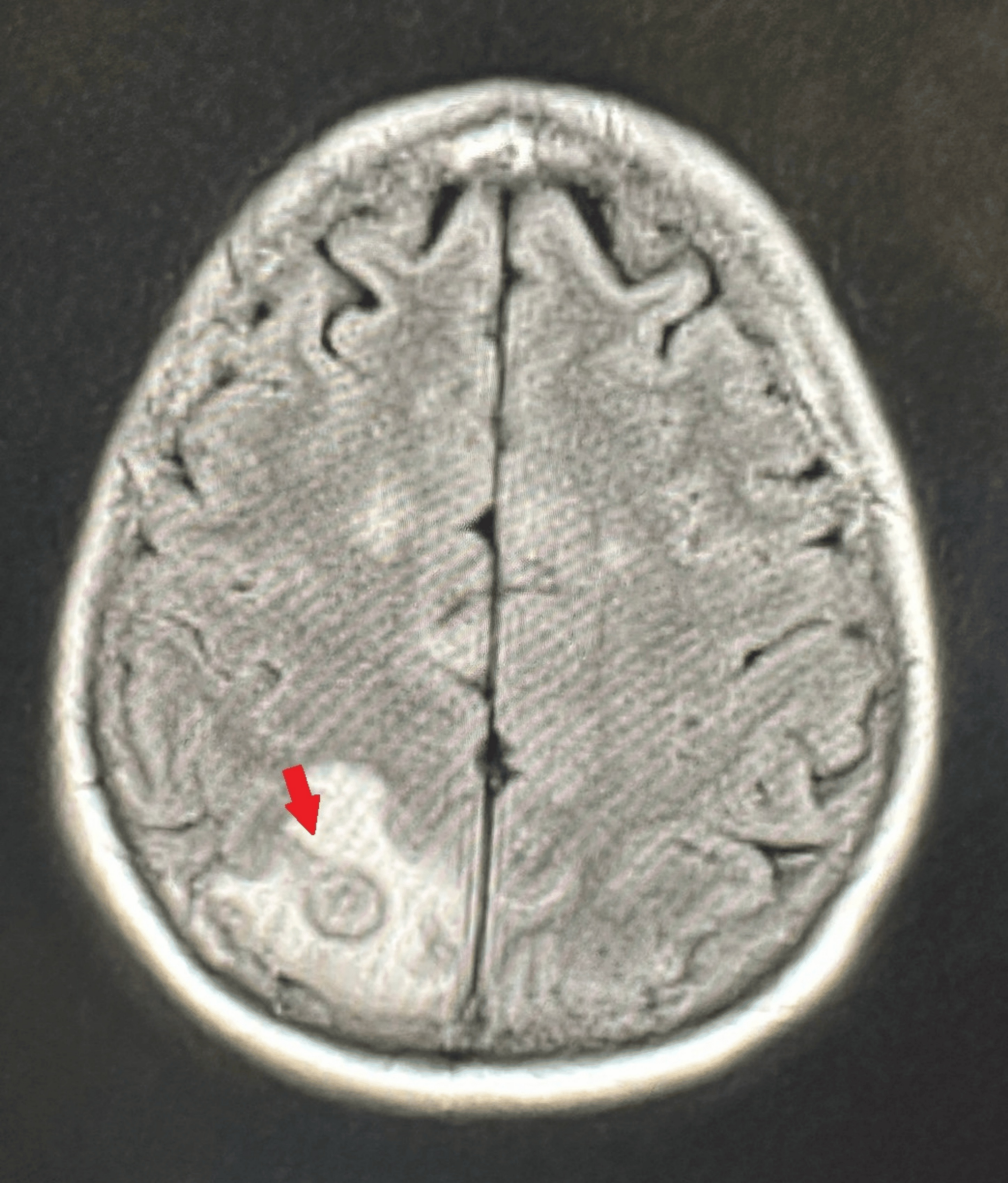
T1/T2: multiple lesions including midbrain, suggestive of disseminated tuberculomas (red arrow)

**Figure 7:**
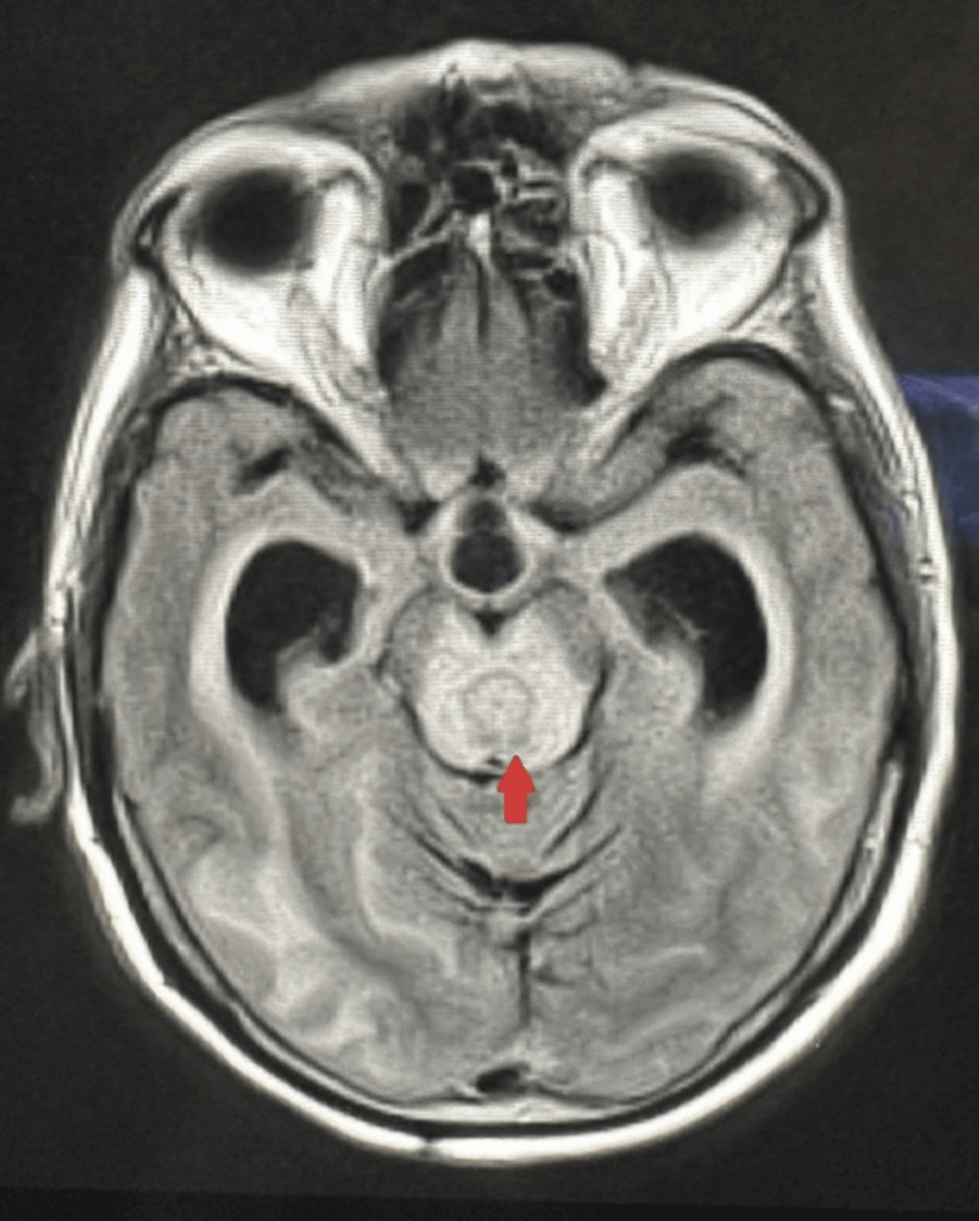
T2: hyperintense midbrain lesion with edema (red arrow)

**Figure 8:**
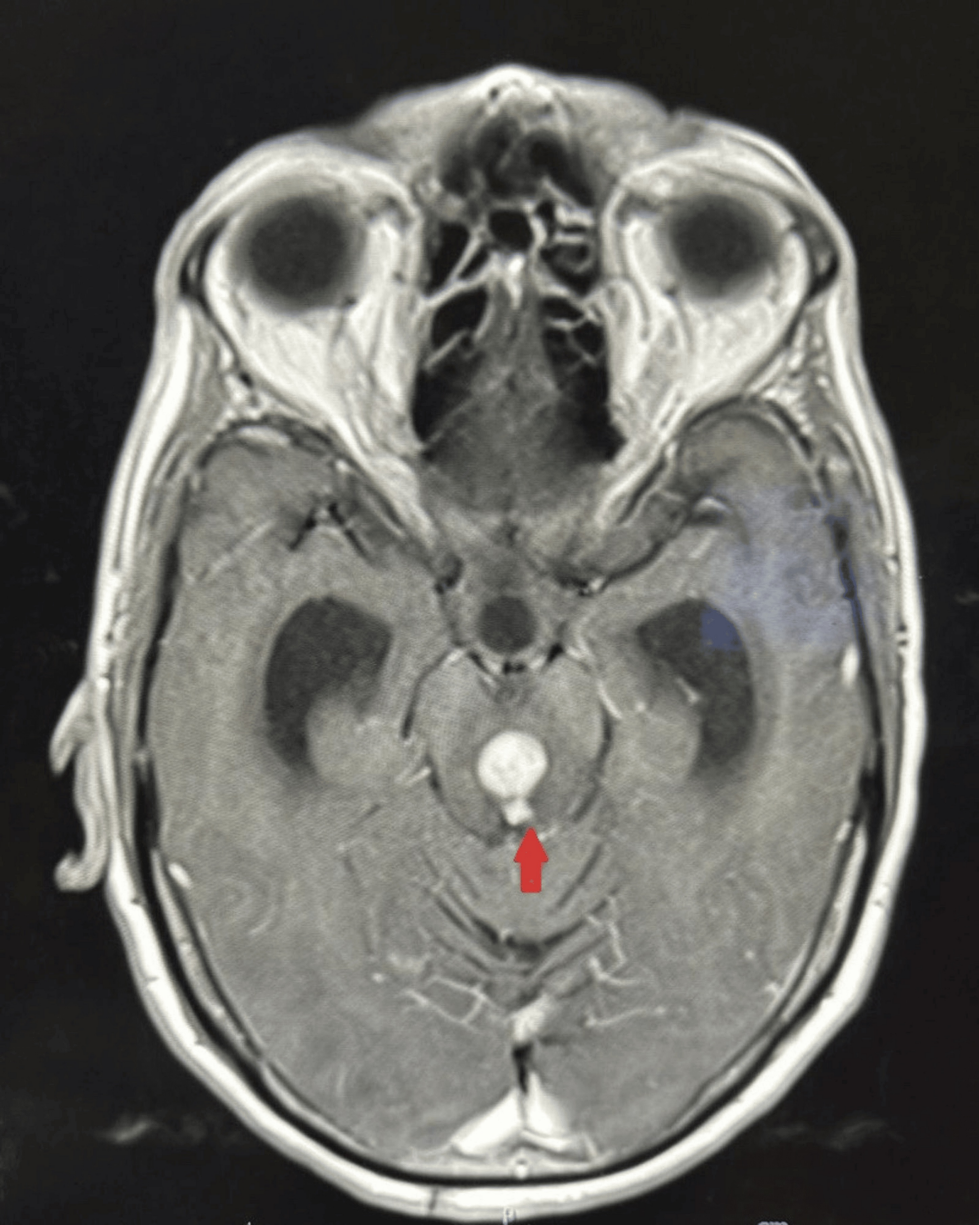
FLAIR: central hypointense lesion with surrounding hyperintensity suggestive of midbrain tuberculoma (red arrow) FLAIR: fluid-attenuated inversion recovery

MRI of the brain with contrast demonstrated multiple ring-enhancing lesions consistent with disseminated tuberculomas distributed in both the supratentorial and infratentorial compartments (Figures 3-6). A focal tuberculoma was identified at the level of the midbrain tegmentum, in the region of the oculomotor nuclear-fascicular complex, providing direct radiological correlation for the bilateral third nerve palsy (Figure 7). T2/FLAIR sequences showed periventricular hyperintensities suggestive of trans-ependymal cerebrospinal fluid (CSF) seepage (Figure 4). Additional FLAIR sequences demonstrated ring lesions with surrounding edema consistent with tuberculoma (Figure 5) and a central hypointense midbrain lesion with surrounding hyperintensity (Figure 8). Mild communicating hydrocephalus was noted with no obstructive component (Figure 3). Meningeal enhancement was present at the basal cisterns, consistent with basal meningitis.

**Figure 9:**
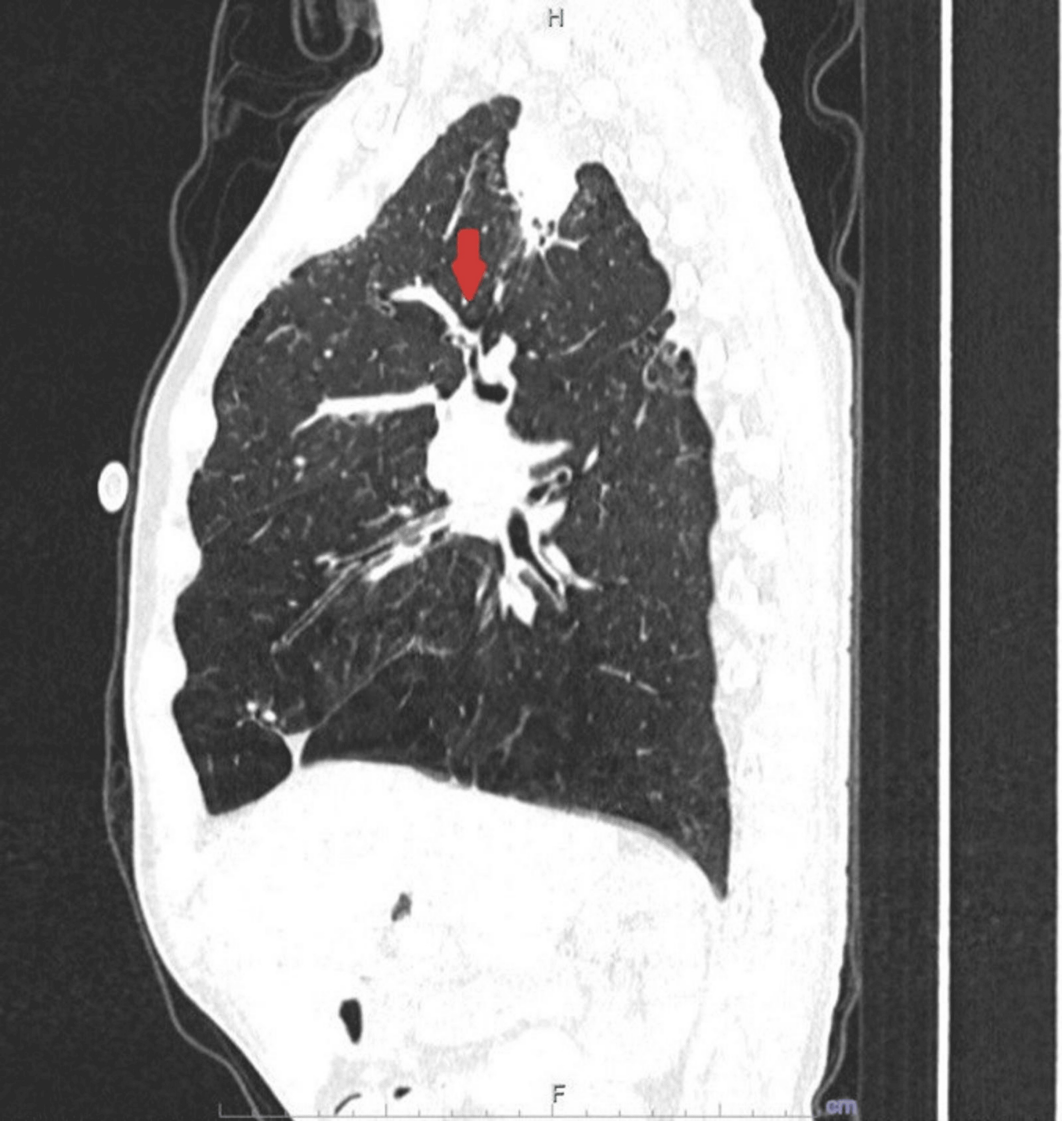
CT of the thorax (coronal): bilateral upper lobe opacities with cavitation (red arrow)

**Figure 10:**
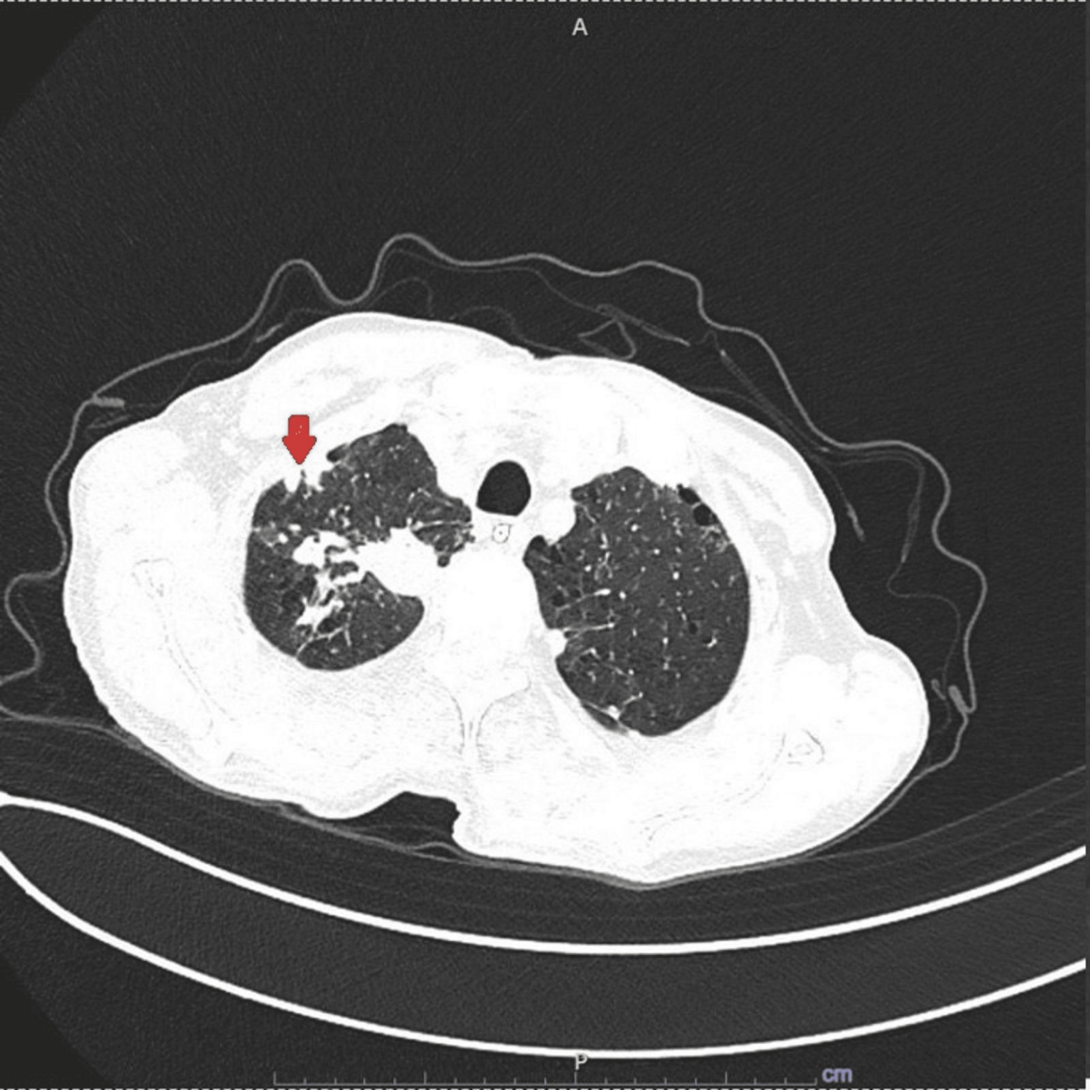
CT of the thorax (axial): thick-walled cavity with surrounding consolidation (red arrow)

High-resolution CT of the thorax revealed bilateral upper lobe nodular opacities with cavitation and associated mediastinal lymphadenopathy, highly suggestive of active pulmonary tuberculosis and indicating a likely hematogenous route of CNS dissemination (Figures 9, 10).

Differential diagnoses are summarized in Table 2.

**Table 2:** Differential diagnoses considered and basis for exclusion ATT: anti-tubercular therapy, CSF: cerebrospinal fluid, ADA: adenosine deaminase, TB: tuberculosis, CNS: central nervous system, HSV: herpes simplex virus, CMV: cytomegalovirus

Differential diagnosis	Reason for exclusion
Fungal meningitis (cryptococcal)	CSF India ink and cryptococcal antigen negative; CSF ADA elevation favors TB
Bacterial meningitis	Subacute course, lymphocytic CSF predominance, and ADA elevation inconsistent
Brainstem stroke	MRI showed ring-enhancing granulomatous lesions, not acute infarction
CNS lymphoma or metastatic disease	No known primary malignancy; imaging pattern and CSF profile inconsistent
Diabetic cranial neuropathy	Typically, unilateral CN III with pupil sparing; bilateral involvement and meningeal signs excludes
Neurosarcoidosis	No hilar lymphadenopathy pattern typical of sarcoid; CT of the thorax showed cavitary disease
Viral encephalitis (HSV and CMV)	Subacute granulomatous imaging pattern, CSF ADA elevation, and pulmonary TB on CT inconsistent with it

Treatment and outcome

A clinical diagnosis of tuberculous meningitis with disseminated cerebral tuberculomas was established. Treatment was initiated promptly with standard four-drug anti-tubercular therapy (isoniazid, rifampicin, pyrazinamide, and ethambutol) along with adjunctive dexamethasone, in accordance with WHO and national TBM treatment guidelines. Insulin therapy was optimized for glycemic control, and serial neurological assessments were performed throughout the hospital course. At the six-week follow-up, the patient showed marked clinical improvement. Bilateral ptosis had partially resolved, with near-complete recovery of right eye movements. Left eye adduction remained mildly restricted. Sensorium improved to GCS 15/15. The patient was discharged on continuation-phase ATT with outpatient ophthalmological and neurological follow-up.

## Discussion

Tuberculous meningitis is characterized by a complex interplay of basal exudative inflammation, obliterative vasculitis, and granuloma formation, leading to diverse neurological manifestations [1]. Cranial nerve palsies occur in a significant proportion of patients and are typically attributed to entrapment within dense basal exudates or ischemic injury secondary to vasculitis [1,5]. Among cranial nerves, the abducens nerve is most frequently involved owing to its long intracranial course, while oculomotor nerve involvement is less common. Bilateral third nerve palsy, however, is distinctly rare and suggests involvement of the oculomotor nuclear complex or fascicles within the midbrain [2,3].

Previous reports have described atypical cranial nerve involvement in tuberculosis: Krishna and Pavuluri [2] documented disseminated tuberculosis presenting with third nerve palsy and aortic aneurysm, while Tangcheewinsirikul et al. [3] highlighted unusual neurological manifestations in systemic tuberculosis. In contrast, the present case demonstrates an unambiguous radiological correlation between a focal midbrain tuberculoma and bilateral ophthalmoplegia, providing a structural anatomical explanation that strengthens diagnostic certainty.

The pupil-sparing nature of the bilateral CN III palsy in this patient deserves specific mention. Classic teaching holds that compressive CN III lesions (e.g., posterior communicating artery aneurysm) typically involve the pupil, as the parasympathetic fibers run superficially. Nuclear or fascicular lesions within the midbrain, however, may spare the pupil if the Edinger-Westphal nucleus is not directly involved. In this case, the midbrain tuberculoma at the oculomotor fascicular complex, rather than compression of the nerve trunk, likely accounts for the pupil-sparing bilateral palsy, consistent with intrinsic midbrain pathology rather than extrinsic compression.

The presence of disseminated tuberculomas suggests hematogenous spread from the pulmonary source documented on thoracic CT. The associated communicating hydrocephalus likely results from impaired CSF resorption at the arachnoid villi due to inflammatory exudates [1,6]. This coexistence of parenchymal, meningeal, and pulmonary disease underscores the multifaceted pathophysiology of disseminated TBM.

A negative Xpert MTB/RIF (CBNAAT) result on CSF, as seen here, should not delay treatment. Published data demonstrate a sensitivity of approximately 45%-70% for Xpert MTB/RIF in CSF for TBM diagnosis; thus, a negative result has limited negative predictive value and cannot exclude the diagnosis when clinical, biochemical, and radiological features are consistent [1]. Treatment should be initiated on clinico-radiological grounds without awaiting microbiological confirmation.

Adjunctive corticosteroid therapy (dexamethasone) is recommended in all cases of TBM regardless of disease severity, as randomized controlled trial evidence demonstrates a reduction in mortality and severe disability through attenuation of the inflammatory response responsible for vasculitis, edema, and cranial nerve entrapment [1]. In addition, comorbid diabetes mellitus, poorly controlled in this patient (HbA1c: 8.2%), represents a significant risk factor for atypical and severe CNS tuberculous disease by impairing cellular immunity and granuloma containment. Optimization of glycemic control is therefore an integral component of management.

## Conclusions

This case illustrates that TBM can present with rare and diagnostically challenging neuro-ophthalmological manifestations. Bilateral oculomotor nerve palsy is an exceedingly uncommon cranial neuropathy in TBM and, when encountered, warrants urgent neuroimaging to identify midbrain or brainstem pathology. In this patient, MRI demonstrated a focal midbrain tuberculoma directly correlating with bilateral third nerve deficits, highlighting the indispensable role of structural neuroimaging in anatomically localizing atypical presentations.
